# Thiazole-Substituted Benzoylpiperazines with Anti-Cancer and Anti-Inflammatory Activities: Comparative Evaluation with Lercanidipine

**DOI:** 10.3390/ijms27177605

**Published:** 2026-08-25

**Authors:** Sevde Nur Biltekin Kaleli

**Affiliations:** Department of Pharmaceutical Microbiology, School of Pharmacy, Istanbul Medipol University, 34810 Istanbul, Türkiye; sevdebltkn@gmail.com or snbiltekin@medipol.edu.tr

**Keywords:** thiazole-substituted benzoylpiperazines, lercanidipine, cisplatin, apoptosis, anti-inflammatory

## Abstract

Lercanidipine has previously been investigated in our laboratory as a reference compound in cancer-related combination studies, providing a comparative framework for the present evaluation of thiazole-substituted benzoylpiperazines. In the present study, we evaluated the cytotoxic, anti-inflammatory, and p38α MAPK-related activities of thiazole-substituted benzoylpiperazine derivatives. The cytotoxic profiles of the derivatives were evaluated in multiple human cancer cell lines using the MTT assay. Compound **29** showed the highest cytotoxic potency among the tested derivatives and was further evaluated in combination with cisplatin and lercanidipine. In SH-SY5Y cells, the combination treatments produced greater cytotoxic effects than the corresponding monotherapies; however, these findings were evaluated as combination effects rather than as evidence of pharmacological synergy. Mechanistic evaluation further revealed that Compound **29** inhibited p38α MAPK enzyme activity and increased caspase-3 and caspase-8 activities. Furthermore, the derivatives exhibited dose-dependent inhibition of TNF-α production, with statistically significant effects at increasing concentrations. Docking into the p38α ATP-binding pocket produced a Vina score of −8.749 kcal/mol and revealed interactions with Tyr35, Lys53, and Asp168. The co-crystallized inhibitor SB203580 showed a score of −9.347 kcal/mol and additionally formed the canonical hinge hydrogen bond with Met109. These complementary *in vitro* and *in silico* findings support p38MAPK as a plausible molecular target of Compound **29**. These *in vitro* and *in silico* findings suggest that p38α MAPK may represent a potential molecular target of Compound **29**. Overall, the findings demonstrate the cytotoxic and anti-inflammatory activities of thiazole-substituted benzoylpiperazines and identify Compound **29** as a potent member of this series for further investigation *in vitro*.

## 1. Introduction

Intracellular calcium (Ca^2+^) homeostasis serves as a fundamental regulatory mechanism, functioning as a versatile secondary messenger that governs a wide array of vital cellular processes, including gene expression, membrane excitability, and the complex cascades of cell proliferation and apoptosis [1]. Under physiological conditions, Ca^2+^ levels are maintained within a strictly narrow range through the coordinated action of various ion channels, pumps, and exchangers located on the plasma membrane and organelle surfaces [2]. However, recent evidence underscores that the deregulation of this calcium signaling pathways is a hallmark of oncogenic transformation, where aberrant expression or activity of calcium-handling proteins promotes tumor survival and facilitates metastasis [3]. Specifically, the sustained elevation or localized oscillations of cytosolic Ca^2+^ can activate downstream signaling effectors that bypass normal growth constraints and induce resistance to programmed cell death [4,5]. Given the central role of calcium signaling in cellular survival and caspase activation, targeting the components of the calcium toolkit has therefore been proposed as a potential strategy for modulating cancer cell responses to conventional chemotherapeutic agents [6].

Lercanidipine, a third-generation 1,4-dihydropyridine calcium channel blocker, was included in the present study as a reference compound based on our previous observations of its biological activity in combination with cisplatin [7,8]. Beyond its established clinical efficacy in managing systemic hypertension and providing end-organ protection, accumulating evidence indicates that the chemical scaffold of DHPs endows them with a broad spectrum of pleiotropic properties, including antioxidant, anti-inflammatory, and anti-apoptotic activities [9,10,11,12]. In the context of oncological research, voltage-gated calcium channels have been shown to be frequently aberrantly expressed in various tumors, where they actively sustain the intracellular calcium gradients required for malignant cell proliferation and survival [8,13,14]. Nevertheless, the present study does not investigate calcium-channel activity or establish a direct pharmacological relationship between lercanidipine and the thiazole-substituted benzoylpiperazines. Rather, lercanidipine was included as a reference compound to facilitate comparative evaluation of cellular responses.

In the strategic design of multi-target directed ligands, the structural hybridization of privileged heterocyclic cores has emerged as a powerful methodology to overcome the limitations of single-target therapies. Among these frameworks, the five-membered thiazole ring represents an exceptionally versatile pharmacophore that has been incorporated into numerous bioactive molecules and has been associated with diverse biological activities, primarily by establishing crucial hydrogen-bonding networks and electrostatic interactions within the ATP-binding pockets of receptor tyrosine kinases such as Vascular Endothelial Growth Factor Receptor (VEGFR) and Epidermal Growth Factor Receptor (EGFR) [15,16]. Concurrently, the incorporation of a piperazine moiety—specifically in the form of a benzoylpiperazine spacer—is a highly valued approach in medicinal chemistry; due to its optimal basicity and conformational flexibility, the piperazine scaffold can contribute to aqueous solubility and molecular recognition and can serve as a structural linker [17]. From an exploratory drug discovery perspective, the scaffold features a central piperazine linker connected to peripheral aromatic rings, offering a flexible structural framework that was evaluated alongside lercanidipine as a benchmark drug in cell-based assays. It should be noted that the present study does not establish quantitative structural, electrostatic, or three-dimensional shape similarity between these compounds and lercanidipine. Accordingly, lercanidipine was used as a pharmacological reference for comparative biological evaluation rather than as a structural template for Compound **29**. While the chemical synthesis and initial neuroprotective profile of this thiazole-substituted benzoylpiperazine library were previously reported by our group [18], the structural features of these derivatives—namely the central flexible piperazine core coupled with peripheral heterocyclic rings—provide a basis for further biological investigation. In modern drug discovery, phenotypic drug repositioning of established chemotypes against distinct pathological targets provides a valuable avenue for identifying unanticipated biological activities. Accordingly, extending the biological evaluation of this scaffold to cancer and inflammation-related cellular models provides an exploratory framework for investigating its effects on cancer cell viability, apoptosis-associated signaling, and pro-inflammatory responses.

Based on this rationale, the present study aimed to characterize the cytotoxicity and anti-inflammatory activities of thiazole-substituted benzoylpiperazine derivatives and to compare selected cellular responses with those observed for lercanidipine. We hypothesized that the combination of the thiazole and benzoylpiperazine structural motifs could confer biologically relevant activity against cancer cells and pro-inflammatory responses. The study further sought to investigate whether the biological effects of the most active derivative were associated with modulation of p38α MAPK activity and caspase-3 and caspase-8 activities.

## 2. Results

### 2.1. Chemistry and Structural Diversity of the Synthesized Compounds

To monitor the biological outcomes and evaluate the structure–activity relationship (SAR) trends of the synthesized series, a compound library consisting of 66 thiazole-substituted benzoylpiperazine derivatives was utilized in this study. The chemical synthesis, characterization, and structural elucidations of these derivatives were previously reported in detail by our group [18]. The molecular library encompasses systematic variations at two distinct aromatic regions of the core scaffold, achieved through the introduction of various electron-donating or electron-withdrawing substituents at the R and R′ positions. For clarity, the core chemical framework, the substituent patterns, and the corresponding numerical designations of all investigated compounds (**1**–**66**) are summarized in Table 1.

### 2.2. In Vitro Cytotoxicity Screening of Thiazole-Substituted Benzoylpiperazine Derivatives

The antiproliferative activity of the synthesized thiazole-substituted benzoylpiperazine derivatives (**1**–**66**), whose chemical synthesis and structural characterization were previously reported by our group [18], was evaluated *in vitro* against four human cancer cell lines: SH-SY5Y (neuroblastoma), PC3 (prostate), MCF7 (breast), and U87MG (glioblastoma. The immortalized human embryonic kidney cell line HEK293 was included as a non-cancerous control cell line to provide a preliminary assessment of selectivity. Lercanidipine and cisplatin served as reference compounds. The screening results, expressed as mean IC_50_ values (µM), are summarized in Table 2, whereas the corresponding Selectivity Index (SI) values, calculated as IC_50(HEK293)_/IC_50(Cancer Line)_, are presented in Table 3 to characterize the relative selectivity of the compounds toward cancer cells.

The screening demonstrated distinct cell-line specific profiles, with the neuroblastoma line (SH-SY5Y) showing the highest overall susceptibility to the evaluated compounds. Conversely, the glioblastoma line (U87MG) generally exhibited lower sensitivity to the tested compounds, with higher concentrations being required to reduce cell viability.

The inclusion of a fluorine atom at the para-position of the benzoyl ring was associated with favorable antiproliferative activity in several members of the series. Compound **29** was identified as the most potent compound, demonstrating an IC_50_ value of 2.23 ± 0.95 µM against SH-SY5Y cells, which represents an approximately 15.7-fold lower IC_50_ value than that of lercanidipine (35.03 ± 1.34 µM). Concurrently, Compound **29** exhibited relatively low cytotoxicity toward HEK293 cells, with an IC_50_ value of 98.10 ± 2.91 µM, corresponding to a Selectivity Index (SI) of 43.99 for SH-SY5Y cells. Other para-fluorinated analogues, such as Compounds **7** and **18**, also showed favorable antiproliferative and selectivity profiles.

Derivatives carrying a para-nitro group, particularly Compounds **11**, **22**, **33**, **44**, **55**, and **66**, showed low-micromolar IC_50_ values across the evaluated cancer cell lines. In contrast, these nitro-functionalized structures also exhibited comparatively lower IC_50_ values in HEK293 cells, ranging from 44.67 to 55.01 µM. As shown in Table 3, this relatively limited separation between cancer-cell and HEK293 IC_50_ values resulted in lower SI values compared with several fluorinated analogues.

Substitutions with methyl or methoxy groups on the benzoyl core resulted in moderate antiproliferative activity profiles. Within this sub-series, para-substituted variants like Compound **3** and Compound **5** showed against SH-SY5Y cells (9.3 µM and 13.90 µM) while exhibiting toward the HEK293 line (88.27 µM and 98.53 µM). Additionally, a para-methoxy arrangement on the piperazine phenyl ring (Compounds **24**–**27**) was generally associated with more favorable antiproliferative and selectivity profiles than ortho- or meta-substituted analogues.

The lowest cytotoxicity profiles and less favorable selectivity indices were observed in derivatives that were either unsubstituted on the benzoyl core (**1**, **12**, **23**, **34**, **45**, and **56**) or carried bulky chlorine atoms. For the chlorinated derivatives, particularly the ortho-chloro variants (Compounds **35**–**38**), the larger steric bulk of the chlorine atom was hypothesized to potentially hinder effective interactions, which might have resulted in reduced anti-cancer efficiency despite their low toxicity in HEK293 cells.

Among the synthesized derivatives, Compound **29** was selected for further mechanistic investigation based on its favorable *in vitro* antiproliferative and selectivity profile. Notably, it demonstrated the most potent cytotoxic efficacy against the SH-SY5Y cancer cell line while simultaneously exhibiting relatively low cytotoxicity toward HEK293 cells. This combination yielded an SI of 43.99 for SH-SY5Y cells, identifying Compound **29** as the most active compound in the present *in vitro* screening panel and supporting its selection for further mechanistic evaluation. Subsequent experiments therefore focused on Compound **29** and examined its effects in combination with reference anticancer agents, caspase activity, p38α kinase activity, and LPS-induced TNF-α production in a macrophage model.

### 2.3. Enhanced Efficacy of Compound ***29*** in Combination with Cisplatin and Lercanidipine

To explore the antiproliferative effects of Compound **29** in combination with reference compounds *in vitro*, fixed-ratio combinations of Compound **29** with cisplatin and lercanidipine were evaluated in SH-SY5Y, PC3, MCF7, and U87MG cell lines. The combination regimens were prepared based on the respective monotherapy IC_50_ values of the individual compounds, followed by serial dilution to determine the IC_50_ values of the combination treatments. The resulting combination IC_50_ values are summarized in Table 4.

As shown in Table 4, the **29** + lercanidipine and **29** + cisplatin combinations generally exhibited lower IC_50_ values than the corresponding monotherapies across the tested cell lines. For the **29** + lercanidipine combination, IC_50_ values were 1.31, 9.99, 21.11, and 18.86 µM in SH-SY5Y, PC3, MCF7, and U87MG cells, respectively. The 29 + cisplatin combination yielded IC50 values of 0.64, 5.41, 8.29, and 11.06 µM, respectively. In contrast, the lercanidipine + cisplatin combination did not consistently produce lower IC_50_ values than both corresponding monotherapies, with IC_50_ values of 12.97, 42.11, 61.73, and 27.43 µM in SH-SY5Y, PC3, MCF7, and U87MG cells, respectively.

The triple combination of Compound **29**, lercanidipine, and cisplatin produced the lowest combination IC50 values among the tested combination regimens, with values of 0.41, 2.82, 5.66, and 5.17 µM in SH-SY5Y, PC3, MCF7, and U87MG cells, respectively. These values were lower than the corresponding monotherapy IC_50_ values of the individual compounds in all four cell lines.

Taken together, these findings demonstrate that the fixed-ratio combination regimens containing Compound **29** were associated with lower IC_50_ values under the tested experimental conditions. However, lower combination IC_50_ values alone do not establish pharmacological synergy. Therefore, the combination data are interpreted here as evidence of increased antiproliferative activity under the tested fixed-ratio conditions rather than as definitive evidence of synergism.

### 2.4. Effects of Compound ***29*** on Caspase-3 and Caspase-8 Activities

**Figure 1:**
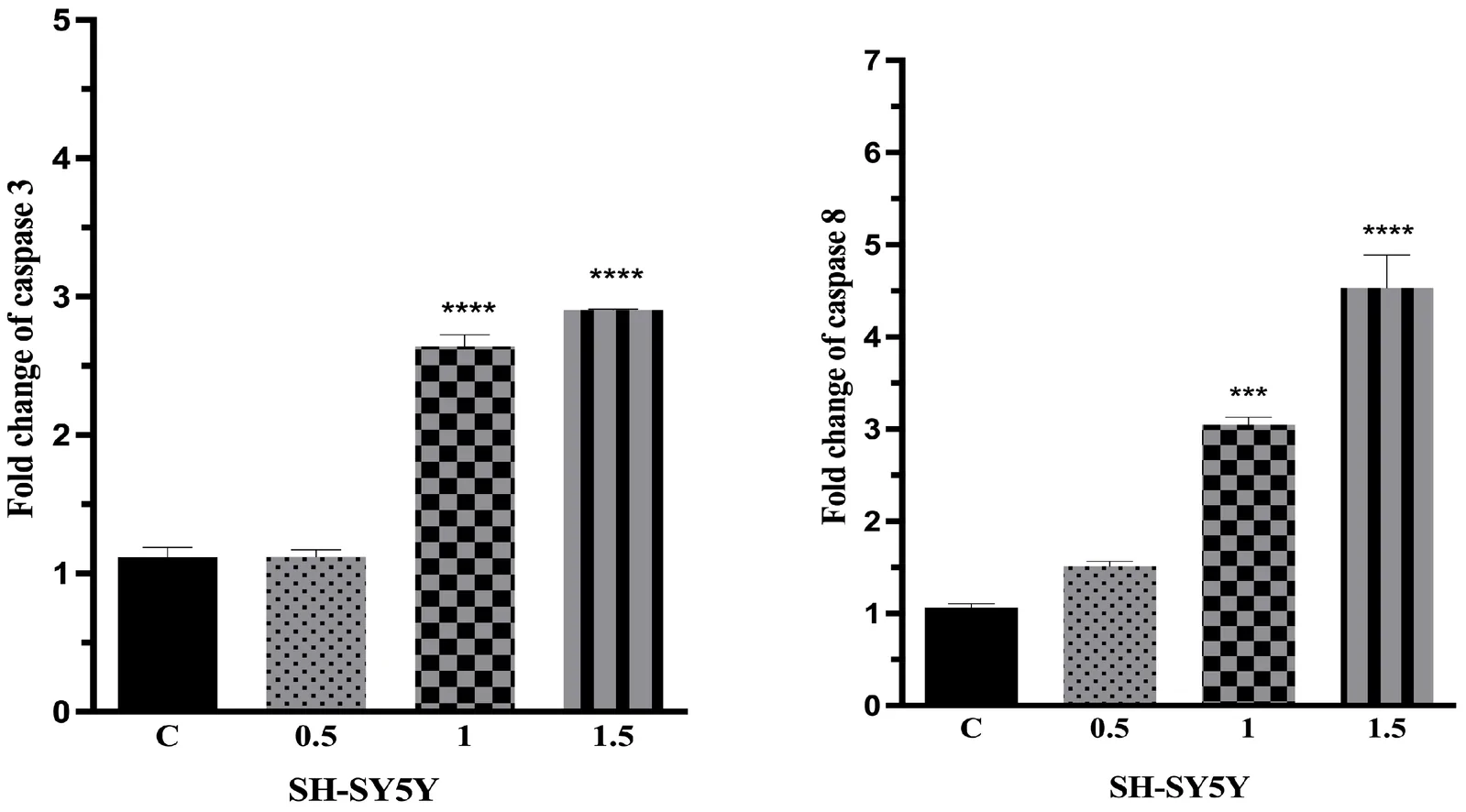
Effects of Compound **29** on caspase-3 and caspase-8 enzyme activities in SH-SY5Y neuroblastoma cells. Cells were treated with increasing concentrations (0.5, 1, and 1.5 µM) of Compound **29**, and fold changes were quantified using commercial ELISA kits. Untreated cells served as the negative control (C). Data are presented as mean ± standard deviation (SD) from three replicates and three independent experiments. Statistical significance compared to the control group is indicated as *** *p* < 0.001 and **** *p* < 0.0001.

To evaluate the apoptotic pathways triggered by our active entity, we monitored its modulatory impact on caspase-3 and caspase-8 enzymatic activities. For this purpose, SH-SY5Y neuroblastoma cells were exposed to varying concentrations of Compound **29** (0.5 µM, 1 µM, and 1.5 µM) prior to cell lysis. The activities of caspase-3 and caspase-8 were subsequently quantified using commercial ELISA-based assay kits, with untreated cells serving as the control group. The relative enzymatic activities of caspase-3 and caspase-8 enzymes in SH-SY5Y cultures following treatment with Compound **29** is illustrated in Figure 1. Compared with the untreated control, treatment with 1.0 and 1.5 µM of Compound **29** significantly increased both caspase-3 and caspase-8 activities, whereas the 0.5 µM treatment did not show a statistically significant difference.

At 1.0 µM, Compound **29** increased caspase-3 and caspase-8 activities to approximately 2.6-fold (**** *p* < 0.0001) and 3.0-fold (*** *p* < 0.001) of the control, respectively. At 1.5 µM, the corresponding activities increased to approximately 2.9-fold and 4.5-fold, respectively (**** *p* < 0.0001). These findings indicate that Compound **29** increased caspase-3 and caspase-8 enzymatic activities under the experimental conditions used.

### 2.5. In Vitro Inhibition of p38MAPK Kinase Activity by Compound ***29***

To investigate whether Compound **29** directly inhibits p38α MAPK kinase activity, a cell-free biochemical kinase assay was performed using a commercial ADP-Glo™ assay. Compound **29**, lercanidipine, and the reference p38α inhibitor SB203580 were evaluated at a fixed concentration of 20 µM under the same assay conditions.

**Figure 2:**
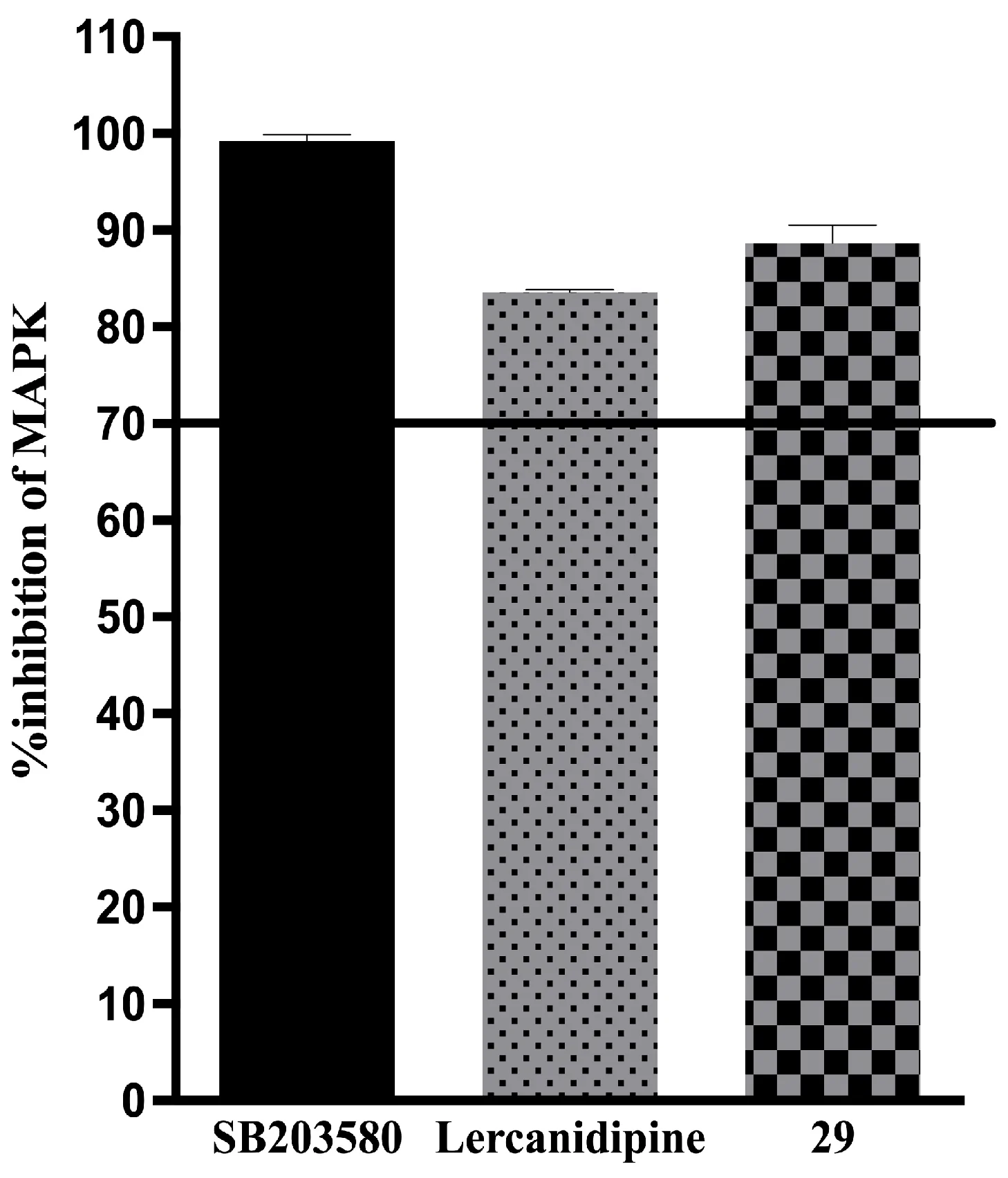
Inhibitory effect of Compound **29** against the p38MAPK enzyme. The biochemical screening was performed under cell-free conditions at a fixed concentration of 20 µM for both lercanidipine and Compound **29**. SB203580 was utilized as the reference p38MAPK inhibitor. Data represent the percentage of enzyme inhibition (%) and are expressed as mean ± standard deviation (SD) from three independent experiments (*n* = 3).

Compound **29** inhibited p38α MAPK kinase activity by 88.63%, whereas lercanidipine produced 83.60% inhibition at the same concentration (Figure 2). These findings demonstrate that Compound **29** can inhibit p38α kinase activity in the cell-free biochemical assay used in this study.

The biochemical inhibition observed in this assay provides experimental support for an interaction with p38α kinase; however, the present data do not establish the cellular mechanism responsible for the antiproliferative effects of Compound **29**.

### 2.6. Dose-Dependent Anti-Inflammatory Activity: Inhibition of TNF-α

To assess the anti-inflammatory activity of Compound **29**, RAW 264.7 macrophages were evaluated for LPS-induced TNF-α production. Cell viability was first assessed under the same treatment conditions to determine whether changes in TNF-α production could be attributed to substantial cytotoxicity. At the highest concentration tested (1.5 µM), Compound **29** maintained cell viability at 89.62% of the untreated control, indicating that the observed reduction in TNF-α production at this concentration was not accompanied by substantial loss of cell viability.

**Figure 3:**
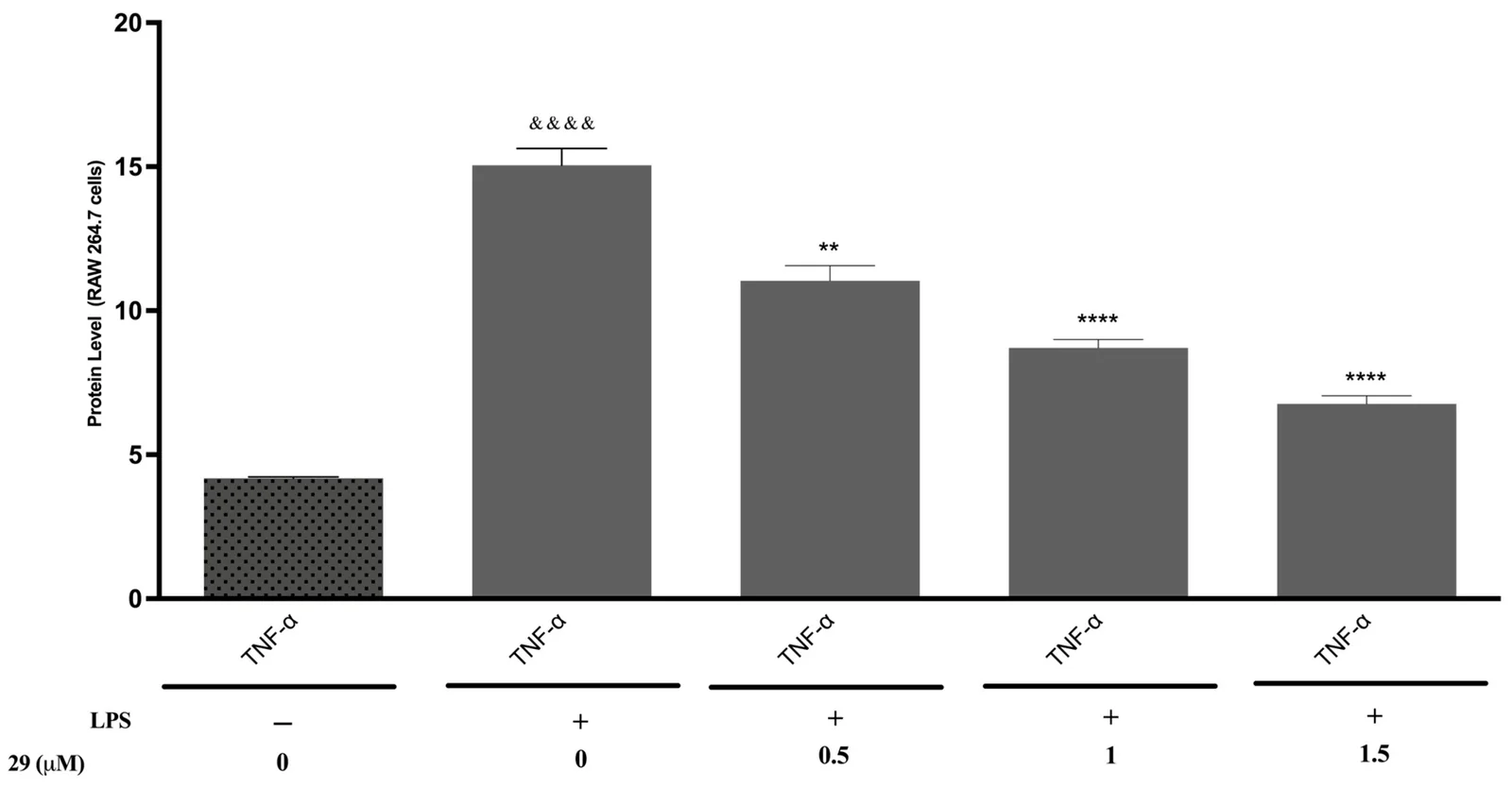
Inhibitory effect of Compound **29** on LPS-induced TNF-α concentration in the culture supernatant in RAW 264.7 macrophage cells. Cells were primed with lipopolysaccharide (LPS, 100 ng/mL) prior to treatment with varying concentrations of Compound **29** (0.5, 1, and 1.5 µM) for 24 h. Effect of Compound **29** on RAW 264.7 cell viability under the conditions used for the TNF-α assay. Unstimulated cells (LPS-, Compound **29** 0 µM) served as the baseline control. Data are presented as mean ± standard deviation (SD) from three independent biological experiments, with each condition performed in technical triplicates (*n* = 3). ^&&&&^ *p* < 0.0001 indicates a statistically highly significant difference compared to the unstimulated control group; ** *p* < 0.01 and **** *p* < 0.0001 indicate significant differences compared to the LPS-only treated group.

RAW 264.7 macrophages were then exposed to Compound **29** at 0.5, 1.0, and 1.5 µM under the LPS stimulation protocol described in the Materials and Methods, and TNF-α concentrations in the culture supernatants were quantified using a commercial ELISA kit. LPS stimulation produced a significant increase in TNF-α production compared with unstimulated control cells (^&&&&^ *p* < 0.0001). Compound **29** reduced LPS-induced TNF-α production in a concentration-dependent manner, with statistically significant reductions observed at the tested concentrations as indicated in Figure 3.

At 0.5 µM, TNF-α levels decreased to approximately 11.0 units (** *p* < 0.01), whereas treatment with 1.0 and 1.5 µM reduced TNF-α levels to approximately 8.7 and 6.7 units, respectively (**** *p* < 0.0001 for the highest concentration). Together, these findings indicate that Compound **29** suppresses LPS-induced TNF-α production in RAW 264.7 macrophages under the experimental conditions used.

### 2.7. In Silico p38 MAPK Docking Studies

Validation of the docking protocol was performed by redocking the co-crystallized ligand, yielding an RMSD of 0.526 Å between the crystallographic and predicted binding poses.

**Figure 4:**
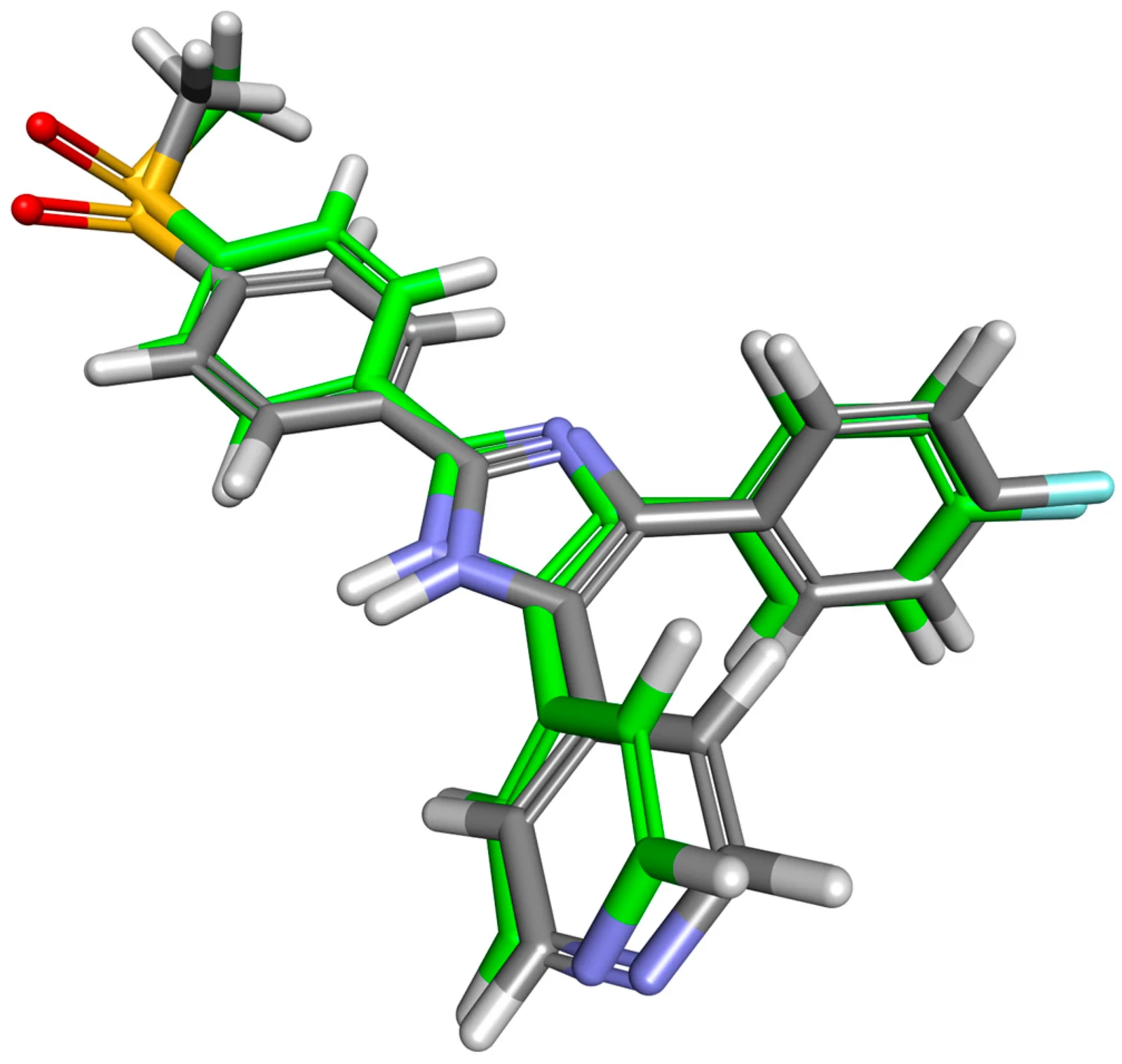
Superposition of the crystallographic binding pose of the co-crystallized ligand SB203580 and its redocked pose within the ATP-binding pocket of p38α MAPK (PDB ID: 1A9U). Carbon atoms of the crystallographic and redocked poses are shown in gray and green, respectively.

**Figure 5:**
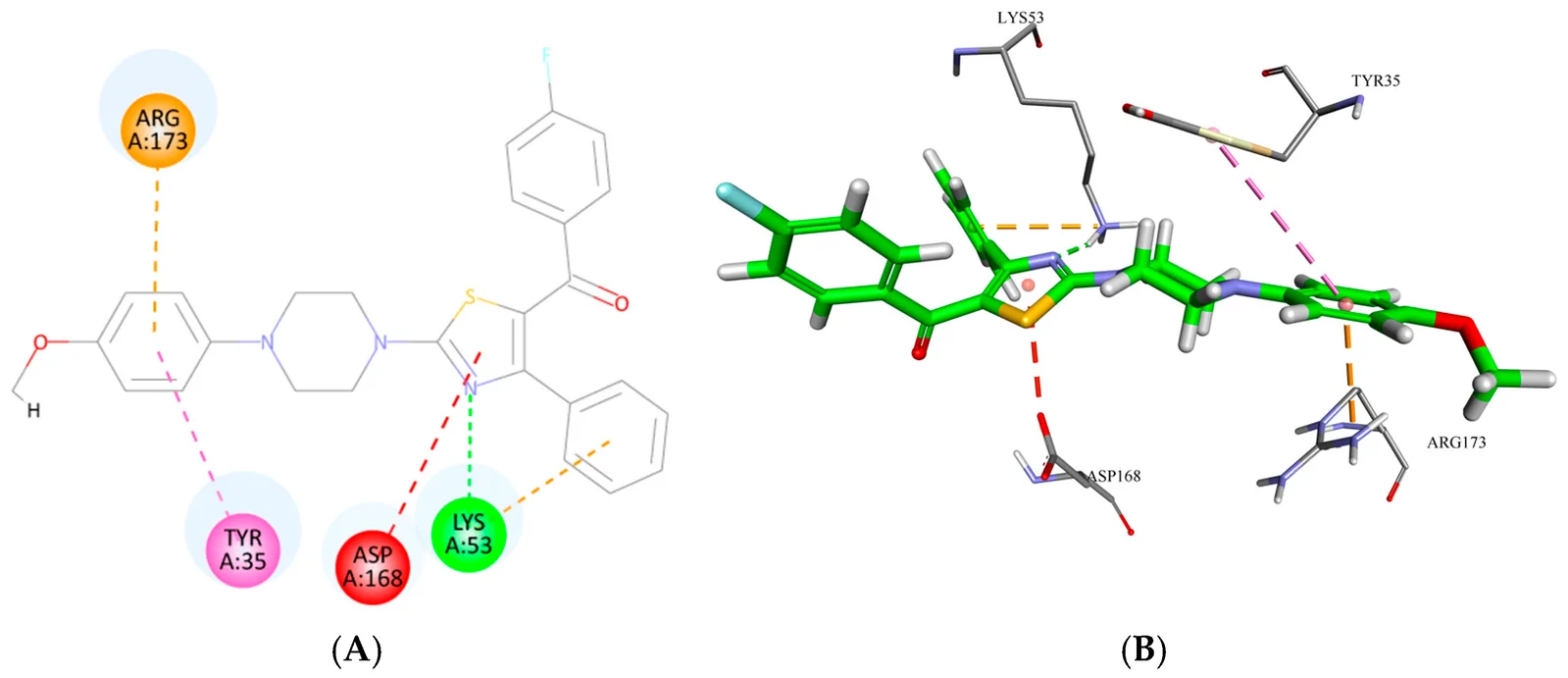
Predicted binding mode of Compound **29** within the ATP-binding pocket of p38α MAPK. (**A**) Two-dimensional representation of the protein–ligand interactions. (**B**) Three-dimensional representation of Compound **29** within the active site. The predicted interactions involve Tyr35, Lys53, Asp168, and Arg173.

Molecular docking within the ATP-binding pocket of p38α MAPK (PDB ID: 1A9U) yielded a Vina score of −8.749 kcal/mol for Compound **29**, close to that obtained for the co-crystallized inhibitor SB203580 (−9.347 kcal/mol). Compound **29** established π–π stacking with Tyr35, π–cation and hydrogen-bond interactions with Lys53, and a π–anion interaction with Asp168. SB203580 reproduced these major contacts and additionally formed the canonical hinge hydrogen bond with Met109 (Figure 4). An additional π–cation contact with Arg173 was predicted; however, this interaction was not considered a primary determinant of binding (Figure 5). These findings support a plausible interaction of Compound **29** with the p38α MAPK ATP-binding pocket, consistent with its *in vitro* inhibitory profile.

## 3. Discussion

The structural composition of these derivatives—combining a flexible central piperazine linker with peripheral heterocyclic architectures—was evaluated in relation to their observed antiproliferative and anti-inflammatory activities. In this study, a molecular series of 66 thiazole-substituted benzoylpiperazine compounds was evaluated through an exploratory phenotypic drug discovery approach, utilizing lercanidipine as a reference compound for comparative biological profiling. While the chemical synthesis and initial neuroprotective profiles of this 66-member library were documented by our group in 2018 [18], extending this scaffold into oncology is grounded in the strategic paradigm of phenotypic drug repositioning. The structural composition of these derivatives—combining a flexible central piperazine linker with peripheral heterocyclic architectures—together with the established biological activities of the reference compound and the broader literature on cancer-cell signaling, provided a basis for exploring their capacity to affect cancer cell proliferation, apoptosis-associated responses, and inflammatory signaling. It is important to emphasize that the present study does not establish quantitative electrostatic or three-dimensional shape similarity between Compound **29** and lercanidipine. Lercanidipine was included as a reference compound for comparative biological evaluation, and no structural or pharmacological equivalence between the two compounds is inferred from the present data. Quantitative ESP and three-dimensional shape analyses would be required to investigate such similarity and therefore represent a direction for future computational studies.

Intracellular calcium (Ca^2+^) signaling is involved in multiple cellular processes relevant to cancer biology, including cell proliferation and survival. Voltage-gated calcium channels (VGCCs) are important regulators of intracellular calcium influx and cellular signaling [2,19,20,21,22,23,24]. However, the present study did not directly investigate calcium-channel expression, calcium influx, electrophysiological activity, or calcium-dependent signaling in Compound **29**-treated cells. Therefore, the biological effects observed here should not be interpreted as evidence of calcium-channel modulation by Compound **29**. Furthermore, lacidipine has been shown to possess the potential to inhibit the viability and proliferation of ovarian cancer stem cells by inducing apoptosis [25]. Taken together, these data suggest that L-type calcium channels and their associated calcium homeostasis regulators may represent relevant biological targets in oncology [19]. Driven by the necessity to effectively modulate these intricate oncological targets, contemporary drug discovery strategies heavily rely on the rational design of small molecules featuring privileged heterocyclic frameworks.

When the literature is reviewed, it is observed that the piperazine ring is an indispensable core scaffold in the structural modification of natural products and the development of favorable pharmacological agents [26]. Likewise, it is also known that piperazine derivatives can strongly block enzyme and receptor systems that regulate tumor growth and survival signaling pathways [27]. Conducted structure–activity and translational medicine studies confirm that piperazine derivatives offer a rational therapeutic platform in antiproliferative mechanisms of action, significantly increasing binding strength and selectivity by establishing strong interactions with acidic residues in the active sites of enzymes, particularly kinases [17]. Similarly, functional structures containing a thiazole ring are also accepted as priority therapeutic targets and focuses of bioisosteric modification in modern oncology research [28,29]. The profound pharmacological relevance of these individual heterocyclic scaffolds underscores the necessity of merging privileged fragments to achieve superior therapeutic efficacy.

Rational drug design approaches in medicinal chemistry are based on the hybridization of bioactive pharmacophores under the same molecular framework, and the combination of heterocyclic cores constitutes a fundamental strategy for enhancing anticancer activity. Several piperazine-based commercial drugs possessing this cyclic ring system have been approved for cancer treatment. In clinical oncology practice, the inclusion of the piperazine scaffold within the structures of FDA-approved agents for breast cancer treatment such as Olaparib, Abemaciclib, and Palbociclib, alongside commercial drugs like Rociletinib used in lung cancer and Imatinib which demonstrates therapeutic success in acute lymphoblastic leukemia treatment, confirms the importance of this ring [30,31]. The structural rationales behind positioning the piperazine ring as a fundamental component in anticancer drug designs include the ring’s basic character, its conformational flexibility, its ability to modulate molecular target binding properties, and its capacity to function among hybrid structures both as a solubility-optimizing motif and as an ideal flexible linker. Therefore, the combination of the piperazine flexible linker scaffold with the thiazole ring (piperazine-based thiazole hybrids) offers a potentially useful medicinal chemistry strategy for exploring antiproliferative activity by leveraging pharmacophore complementarity [32]. In vitro cytotoxicity screenings in the literature indicate that such piperazine–thiazole hybrid structures produce remarkable antiproliferative effects against different human cancer cell lines, such as hepatoblastoma (HepG2), human colorectal carcinoma (HCT 116), and breast cancer (MCF-7), in low micromolar to low nanomolar ranges [17,32].

Comprehensive phenotypic screenings identified Compound **29** as the most active compound in the present screening panel. Compound **29** exhibited low-micromolar IC_50_ values against aggressive cancer lines such as SH-SY5Y, PC3, MCF7, and U87MG, while demonstrating minimal toxicity in immortalized non-cancerous control HEK293 cells (IC_50_ = 98.10 ± 2.91 μM), thereby capturing a remarkably high selectivity index (SI) against neuroblastoma cells. In the paradigm of targeted drug design, such a differential *in vitro* cytotoxicity profile indicates preferential activity toward the tested cancer cell model relative to the HEK293 cell line. While the reference drug cisplatin induced severe, non-specific toxicity against the control cells (IC_50_ = 3.11 ± 0.98 μM), yielding an unfavorable selectivity index (SI = 0.38), Compound **29** achieved a remarkable selectivity margin (SI = 43.9) toward the SH-SY5Y neuroblastoma model. However, whether this *in vitro* selectivity translates into reduced systemic toxicity remains unknown and requires dedicated pharmacokinetic and toxicological evaluation.

In combination experiments designed to evaluate enhanced antiproliferative activity in cell-based models, the cytotoxic performance of Compound **29** was evaluated alongside reference therapeutics. Rather than relying solely on individual monotherapy profiles, comparative cytotoxicity assessments confirmed marked efficacy enhancements under the tested *in vitro* conditions across all evaluated dual and triple combination regimens incorporating Compound **29**. The combination regimens incorporating Compound **29** produced lower combination IC_50_ values than the corresponding monotherapies under the tested fixed-ratio conditions. However, these findings alone do not establish pharmacological synergy, as a formal synergy analysis was not performed. Notably, the triple combination regimen (Compound **29** + lercanidipine + cisplatin) produced the most substantial reduction in cell viability, particularly in U87MG glioblastoma and SH-SY5Y neuroblastoma models under the tested fixed-ratio conditions.

The experimental combinations involving Compound **29** consistently demonstrated superior antiproliferative potency compared to the standard lercanidipine–cisplatin reference pairing. While the combination of the two standard reference drugs yielded moderate efficacy gains, the binary combination of Compound **29** with cisplatin produced pronounced cytotoxic responses across all tested cell lines. Furthermore, while the binary combination of Compound **29** with lercanidipine showed a lower combination IC_50_ value than the corresponding monotherapies, the highest degree of growth inhibition based on combination IC_50_ values was achieved with the triple combination treatment (Compound **29** + lercanidipine + cisplatin). Overall, these combinatorial profiles suggest that Compound **29** was associated with enhanced antiproliferative activity when combined with the reference agents under the tested fixed-ratio conditions, as reflected by the lower combination IC50 values. These findings should be interpreted as enhanced combination efficacy rather than definitive evidence of pharmacological synergy.

One of the most fundamental mechanisms of action of chemotherapeutic agents in arresting tumor progression is the induction of cellular apoptosis, and apoptosis induction is a key step in the anticancer performance of piperazine-thiazole derivatives [17]. Within cellular signaling cascades, the activation of caspase proteins is triggered, steering tumor cells toward caspase-dependent apoptotic signaling and potentially contributing to apoptotic cell death [32].

The association between this potent antiproliferative effect of Compound **29** and the activity of caspases involved in apoptotic cascades was established. Caspase enzymatic assays revealed that Compound **29** induced a dose-dependent increase in caspase-3 enzymatic activity (2.9-fold) and particularly caspase-8 enzymatic activity (4.5-fold) in SH-SY5Y cells. These findings are consistent with involvement of caspase-dependent cell-death processes, but they do not by themselves establish activation of a specific intrinsic or death-receptor-mediated apoptotic pathway. The increase in caspase enzymatic activity observed in the present study is broadly consistent with reports on similar piperazine and thiazole hybrid structures [32].

To assess the inhibitory activity of Compound **29** against p38α kinase independently of cellular uptake and intracellular signaling, we tested p38MAPK enzyme inhibition in a cell-free biochemical assay. At a fixed concentration of 20 µM, Compound **29** demonstrated 88.63% inhibition of p38α MAPK activity. This value is above the inhibition level of 83.60% found for lercanidipine under identical experimental conditions in our group’s previous study [9]. It is known in the literature that the p38MAPK pathway plays a dual role; while it exerts a suppressor effect in early-stage tumors, it directly manages pro-survival signals, drug resistance, and inflammatory cytokine release in advanced-stage and metastatic malignancies [33]. The recombinant p38α kinase assay provided direct biochemical evidence that Compound **29** inhibits p38α catalytic activity. Together with the docking analysis, which predicted favorable accommodation of Compound **29** within the ATP-binding pocket, these findings support p38α as a plausible molecular target. However, the biochemical assay does not by itself establish the precise binding mode predicted by docking.

Since tumor progression is directly associated with a pro-inflammatory microenvironment that nourishes angiogenesis and immune evasion processes [34], the anti-inflammatory capacity of Compound **29** was investigated in an LPS-induced RAW 264.7 macrophage model. The experiment showed that LPS stimulation increased cytokine production from basal levels to approximately 15 units, inducing inflammation, and Compound **29** suppressed the dose-dependent inflammatory response. At a dose of 1.5 µM, it successfully reduced TNF-α protein levels in LPS-stimulated macrophages, suggesting an ability to attenuate pro-inflammatory cytokine release in cell-based macrophage models (6.7 units).

A general evaluation of the synthesized series suggests that the biological profile of the compounds is remarkably influenced by the electronic and lipophilic properties of the peripheral substituents. The prominent dual anti-cancer and anti-inflammatory potential observed for Compound **29** might be attributed to a favorable combination of the 4-OCH_3_ group at the R position and the 4-F atom at the R′ position. The presence of the electron-donating para-methoxy (4-OCH_3_) moiety on the piperazine-linked phenyl ring is considered to modulate the local electron density, which could potentially facilitate weaker non-covalent or dipole–dipole interactions within the possible biological target zones. Concurrently, the incorporation of a para-fluorine (4-F) atom on the benzoyl core appears to offer a balanced steric and electronic environment. In medicinal chemistry, the introduction of a fluorine atom, particularly at the para position of aromatic rings, is frequently utilized to optimize physicochemical properties, improve membrane permeability, or enhance metabolic stability by hindering potential oxidative degradation [35,36]. Systematic comparison across the 66-member series reveals that positional shifts and alternative halogenation patterns markedly alter this bioactivity profile. Specifically, derivatives incorporating substituent modifications at the ortho or meta positions generally exhibited diminished antiproliferative efficacy. This reduction in activity may be related to steric effects that could influence the molecular conformation and interactions with biological targets. Furthermore, replacing the compact, highly electronegative 4-F atom with bulkier halogen substituents, such as chlorine or bromine, or introducing multiple halogenations, did not reproduce the prominent potency of Compound **29**. The increased atomic radius and altered lipophilicity associated with heavier halogens likely impose steric constraints that impede tight accommodation into enzyme binding clefts.

Therefore, the specific combination of an electron-donating group on one side and a strategically placed halogen on the other may contribute to the observed biological profile of Compound **29**. This structural pattern may contribute to the observed cytotoxic activity of Compound **29** against SH-SY5Y cells and its notable anti-inflammatory profile, while maintaining a favorable selectivity index compared to several other derivatives in the series. To elucidate the structural and binding basis of these biological observations at the atomic level, *in silico* molecular docking studies were conducted within the ATP-binding pocket of p38α MAPK (PDB ID: 1A9U). Prior to evaluating the compound, the reliability of the docking protocol was validated by redocking the co-crystallized inhibitor SB203580, which demonstrated excellent pose fidelity with an RMSD value of 0.526 Å (Figure 4). Compound **29** displayed favorable predicted binding within the catalytic pocket, achieving a Vina docking score of −8.749 kcal/mol, which is closely comparable to that of SB203580 (−9.347 kcal/mol). Detailed interaction analysis revealed that Compound **29** engages key active site residues through specific non-covalent contacts: it forms π–π stacking interactions with Tyr35, key π–cation and hydrogen-bonding interactions with Lys53, and a π–anion interaction with Asp168 (Figure 5). These predicted interactions provide a structural hypothesis for the interaction of Compound **29** with the p38α ATP-binding pocket and are consistent with its observed biochemical inhibition.

The present findings establish Compound **29** as an active *in vitro* hit candidate within this thiazole-substituted benzoylpiperazine series. In the hit-to-active optimization framework of medicinal chemistry, translation from promising cell-based efficacy to systemic therapeutics requires a comprehensive profiling of ADMET properties. While the current study focused primarily on elucidating the phenotypic anticancer efficacy, apoptotic mechanisms, and anti-inflammatory target profiles, further progression of this chemotype will depend on establishing its physicochemical stability, aqueous solubility, microsomal clearance, and membrane permeability profiles. Integrating these computational and experimental ADMET parameters will represent a crucial milestone in guiding future structural optimization and defining the translational drug-likeness of this series.

In summary, Compound **29**, a thiazole-benzoylpiperazine hybrid, has demonstrated *in vitro* cytotoxic and anti-inflammatory activities under the experimental conditions used in this study. It also demonstrates biochemical inhibition of p38α MAPK and increased caspase-3 and caspase-8 enzymatic activities in SH-SY5Y cells, together with suppression of TNF-α release in LPS-stimulated macrophages. The biochemical findings provide experimental support for the computationally predicted interaction of Compound **29** with p38α. However, the present findings do not establish that p38α inhibition is responsible for the observed antiproliferative or combination-associated effects. These findings provide a basis for further investigation of whether p38α MAPK inhibition contributes to the observed combination-associated enhancement of cisplatin activity. Taken together, the present findings identify Compound **29** as an active *in vitro* hit within the investigated series and provide a rationale for further evaluation of its antiproliferative, anti-inflammatory, and p38α MAPK-related activities, and subsequent ADMET and *in vivo* studies required to determine its translational potential.

## 4. Materials and Methods

### 4.1. Chemicals and Reagents

The 66 thiazole-substituted benzoylpiperazine derivatives investigated in this study were synthesized and structurally characterized via NMR and mass spectrometry by Prof. Dr. Şeref Demirayak, as previously described in our collaborative research [18]. For the biological assays, Lercanidipine hydrochloride, cisplatin, and 3-(4,5-dimethylthiazol-2-yl)-2,5-diphenyltetrazolium bromide (MTT) were purchased from Sigma-Aldrich (St. Louis, MO, USA). High-glucose Dulbecco’s Modified Eagle Medium (DMEM), RPMI 1640 medium, fetal bovine serum (FBS), and penicillin-streptomycin were obtained from Gibco (San Diego, CA, USA and St. Louis, MO, USA). All other analytical grade solvents and chemicals, including dimethyl sulfoxide (DMSO) and phosphate-buffered saline (PBS), were supplied by Merck (Darmstadt, Germany). Stock solutions of the synthetic derivatives and Lercanidipine were prepared in molecular-biology-grade DMSO and stored at −20 °C, with final solvent concentrations maintained below 0.1% (*v*/*v*) to ensure no interference with cellular viability. The p38α MAPK kinase assay system was obtained from Promega (Madison, WI, USA). Caspase assay kits were purchased from Elabscience (E-CK-A311-E-CK-A312, Wuhan, China). All the other chemicals were obtained from Sigma.

### 4.2. Cell Lines and Culture Conditions

The biological assays were conducted using a diverse panel of human cell lines and a murine macrophage model to evaluate biological activities of the synthesized derivatives and Lercanidipine: HEK293 (human embryonic kidney), PC3 (human prostate adenocarcinoma), SH-SY5Y (human neuroblastoma), U87MG (human glioblastoma), MCF7 (human breast adenocarcinoma) and RAW 264.7 (murine macrophages). All cell lines were obtained from the American Type Culture Collection (ATCC, Manassas, VA, USA). The cells were maintained in their respective growth media: DMEM for HEK293, U87MG and SH-SY5Y, and RPMI-1640 for PC3 and MCF7, each supplemented with 10% (*v*/*v*) heat-inactivated fetal bovine serum (FBS), 2 mM L-glutamine, and a 1% antibiotic-antimycotic solution (100 U/mL penicillin and 100 µg/mL streptomycin). For the MCF7 cell line, the culture medium was additionally supplemented with 0.01 mg/mL (10 µg/mL) bovine insulin to support cell proliferation and maintain the characteristic growth phenotype of this cell line. The RAW 264.7 cell line was maintained in high-glucose DMEM supplemented with 10% FBS and subcultured using cell scrapers within a low passage range (passages 5–15) to preserve functional stability and prevent phenotypic drift, strictly adhering to established passage stability guidelines. Cultivation was carried out in a humidified atmosphere containing 5% CO_2_ at 37 °C. To maintain phenotypic stability and ensure experimental reproducibility, cells were observed daily and subcultured upon reaching 80% confluence using 0.25% trypsin-EDTA (or cell scrapers for RAW 264.7 macrophages). All experiments were performed using cells between passage numbers 5 and 15. Regular screening for mycoplasma contamination was conducted to verify the integrity of the cultures throughout the study period [37,38,39].

### 4.3. In Vitro Cytotoxicity Assay

The cytotoxic potential of the synthesized thiazole-substituted benzoylpiperazine derivatives, as well as the reference drugs lercanidipine and cisplatin, was determined using the colorimetric 3-(4,5-dimethylthiazol-2-yl)-2,5-diphenyltetrazolium bromide (MTT) assay [37,40]. Briefly, HEK293, PC3, SH-SY5Y, U87MG and MCF7 cells in the exponential growth phase were harvested and seeded into 96-well microplates at optimized densities 5 × 10^3^ cells/well for HEK293, PC3, U87MG, and MCF7 and 10^4^ cells/well for SH-SY5Y). The plates were incubated for 24 h to allow for cell attachment and stabilization. Prior to cytotoxicity testing, growth curve optimization experiments were conducted to confirm that these initial seeding densities maintained the cells in exponential growth phase and kept the MTT absorbance signal within the linear dynamic range of the assay throughout the treatment period.

The test compounds were dissolved in DMSO (<1%) and diluted with culture medium to yield a final serial dilution range of 0.1 to 100 µM across 8 concentration points (0.1, 0.5, 1, 2.5, 5, 10, 50, and 100 µM). The cells were subsequently exposed to the test compounds for a treatment duration of 48 h. After the incubation period, the medium was removed, the cells were washed with D-PBS, and 30 μL of MTT stock solution (5 mg/mL in D-PBS) was added to each well and the plates were incubated for an additional 4 h at 37 °C to facilitate the formation of purple formazan crystals by viable cells. The supernatant was then carefully aspirated, and the resulting formazan precipitates were dissolved in 150 µL of DMSO. The absorbance was measured at a wavelength of 570 nm using a microplate reader (Molecular Devices, Sunnyvale, CA, USA).

All assays were conducted as three independent biological experiments, with each condition performed in technical triplicates. The IC_50_ values (the concentration required to inhibit cell viability by 50%) were derived from non-linear regression analysis using a variable-slope dose–response model in GraphPad Prism (version 7.02). Bottom constraints were set to 0% and top constraints to 100%. All reported IC_50_ values represent the mean ± standard deviation (SD). The percentage of cell viability was calculated using the formula below:

Viability (%) = Absorbance_sample_/Absorbance_control_ × 100

### 4.4. Combination Cytotoxicity Analysis

To evaluate the effect of combination treatment, Compound **29** was selected for further combination studies. Based on the individual IC_50_ values determined via the MTT assay, SH-SY5Y, PC3, MCF7, and U87MG cell lines were subjected to treatment regimens. The cells were treated with Compound **29** in combination with lercanidipine and cisplatin, as well as a triple combination involving all three agents at their respective IC_50_ concentrations. The effects of the combination treatments were evaluated by comparing cell viability with the corresponding monotherapy groups. These comparisons were considered measures of combination effects and were not interpreted as formal pharmacological synergy.

### 4.5. Evaluation of Caspase Activities

To evaluate changes in apoptosis-associated caspase activity, the enzymatic activities caspase-3 and caspase-8 were quantified using colorimetric assay kits following the manufacturer’s protocols (Elabscience, E-CKA311-E-CKA312, Wuhan, China). Briefly, cells were treated with Cell Lysis Buffer (50 mM HEPES, pH 7.4, 5 mM CHAPS, 5 mM DTT), incubated on ice for 10 min, and centrifuged at 16,000× *g* for 20 min at 4 °C. Samples were then added to the appropriate wells as specified in the protocol. The protein-containing supernatants were incubated with specific substrates (Ac-DEVD-pNA for Caspase-3 and Ac-IETD-pNA for Caspase-8) at 37 °C for 90 min. The release of p-nitroaniline was measured spectrophotometrically at 405 nm using a microplate reader [37,41,42]. All measurements were expressed as relative fold-change in caspase enzymatic activity compared with untreated control cells.

### 4.6. p38 MAPK Kinase Activity Assay

To assess the inhibitory effect of Compound **29** on p38α MAPK catalytic activity under cell-free conditions, a recombinant human p38α kinase assay was performed using the p38α Kinase Enzyme System (Promega, Madison, WI, USA; Cat. No. V2701) in combination with the ADP-Glo™ Kinase Assay (Promega; Cat. No. V9101), according to the manufacturer’s instructions. The V2701 system contains recombinant active human p38α kinase, a p38α substrate, reaction buffer, and dithiothreitol (DTT).

Briefly, the kinase reaction was performed in white 96-well microplates at room temperature on a plate shaker. Compound **29** was evaluated at a final concentration of 20 µM. Lercanidipine was tested at the same final concentration to permit direct comparison with the reference compound, while SB203580 was included as a reference p38α inhibitor. Inhibitors were pre-incubated with p38α MAP kinase for 15 min at room temperature prior to initiating the reaction. The kinase reaction was initiated by adding the substrate and ATP to achieve final concentrations of 2.5 ng/µL p38α MAPK, 0.2 ng/µL substrate, and 100 µM ATP. The reaction mixture was incubated at room temperature for 60 min.

Following the kinase reaction, ADP-Glo™ Reagent was added to terminate the reaction and deplete remaining ATP, followed by an incubation time of 60 min at room temperature. Subsequently, ADP-Glo™ Kinase Detection Reagent was added and incubated at room temperature for 30 min. The ratio between the kinase reaction, ADP-Glo™ Reagent, and ADP-Glo™ Kinase Detection Reagent was maintained at 1:1:2. Luminescence was measured using a microplate reader (SpectraMax i3, Molecular Devices).

Kinase activity was quantified based on the luminescent signal generated from ADP formed during the kinase reaction. Appropriate controls included an enzyme-containing vehicle control (positive control with p38α MAPK, substrate, and buffer), a negative control containing all reaction components without enzyme (background), and reference inhibitor controls. The percentage inhibition of p38α kinase activity was calculated relative to the vehicle-treated enzyme control. All measurements were performed in three independent experiments, with each condition analyzed in technical replicate.

### 4.7. Measurement of TNF-α Anti-Inflammatory Activity

The anti-inflammatory potential of Compound **29** was evaluated by quantifying the production of the pro-inflammatory cytokines, TNF-α, in a lipopolysaccharide (LPS)-stimulated macrophage model. RAW 264.7 cells were seeded into 96-well microplates at a density of 10^4^ cells/well and allowed to adhere for 24 h at 37 °C in a humidified 5% CO_2_ atmosphere. To initiate the inflammatory response, cells were exposed to LPS at a concentration of 100 ng/mL. Following LPS priming, the culture medium was replaced and the primed cells were treated with various concentrations of Compound **29** (0.5, 1, and 1.5 µM) for a 24 h treatment period.

Following the treatment period, the cell culture supernatants were harvested and clarified by centrifugation at 2000× *g* for 10 min to remove cellular debris. The concentrations of TNF-α in the supernatants were determined using commercially available ELISA kits (Elabscience, Houston, TX, USA) following the manufacturer’s instructions. Briefly, samples and standards were added to the pre-coated microplates and incubated for 90 min at 37 °C. This was followed by successive incubations with the Biotinylated Detection Antibody Working Solution for 1 h and the HRP Conjugate Working Solution for 30 min. After washing steps to remove unbound components, the Substrate Reagent was added, and the chromogenic reaction was allowed to proceed for 15 min in the dark. The reaction was terminated with a Stop Solution, and the absorbance was measured at 450 nm using a microplate spectrophotometer (Molecular Devices, LLC, San Jose, CA, USA). Cytokine levels were calculated based on the linear regression of the standard curves and expressed as percentage inhibition relative to the LPS-only treated control [39,43].

### 4.8. Molecular Docking

For the molecular docking study targeting the p38α MAPK binding pocket, the crystal structure of p38α MAPK co-crystallized with SB203580 (PDB ID: 1A9U) was retrieved from the RCSB Protein Data Bank. The protein structure was prepared using AutoDockTools 1.5.7 [44]. All water molecules were first removed, polar hydrogen atoms were added, and Gasteiger charges were assigned.

Compound **29** was constructed and saved using Avogadro 1.0.2 software [45]. Compound **29** and the co-crystallized ligand extracted from the crystal structure were prepared using AutoDockTools 1.5.7 by adding polar hydrogen atoms, assigning Gasteiger charges, and defining rotatable bonds.

Molecular docking calculations were performed using AutoDock Vina 1.2.7 [46,47]. The grid box was centered on the binding site of the co-crystallized ligand at coordinates x = 2.711, y = 14.693, and z = 28.534 Å, with dimensions of 24 × 24 × 24 Å. The docking parameters were set to an exhaustiveness value of 64, 20 output binding modes, and an energy range of 4 kcal/mol. Visualization of the redocking results and the two- and three-dimensional protein–ligand interactions were performed using BIOVIA Discovery Studio Visualizer 2025 [48].

### 4.9. Statistical Analysis

All experimental procedures were conducted in at least three independent biological experiments, and the resulting data were expressed as the mean ± standard deviation (SD). The normality of the distribution and the homogeneity of variance were verified prior to comparative analyses. Statistical significance between the treatment groups and the respective controls was evaluated using one-way analysis of variance (ANOVA), followed by Tukey’s post hoc test for multiple comparisons. All statistical computations and graphical representations were performed using GraphPad Prism software, version 7.02 (GraphPad Software, La Jolla, CA, USA). A *p*-value of less than 0.05 was considered to indicate a statistically significant difference.

## 5. Conclusions

In conclusion, this study demonstrates the successful development of a series of thiazole-substituted benzoylpiperazine derivatives, with Compound **29** identified as the most active *in vitro* hit for further investigation. By inhibiting cell proliferation and showing activity in a cell-free p38α MAPK biochemical assay, this specific derivative demonstrated a favorable combination of antiproliferative, anti-inflammatory, and p38α MAPK-inhibitory activities under the experimental conditions investigated. Ultimately, the cytotoxicity and anti-inflammatory profile of Compound **29** provides a basis for further investigation of this chemotype rather than establishing its translational or therapeutic potential.

Compound **29** demonstrated substantial inhibition of p38α MAPK activity at the tested concentration and a favorable predicted binding mode within the ATP-binding pocket. The predicted interactions with Tyr35, Lys53, and Asp168 provide a structural hypothesis for its interaction with the p38α MAPK ATP-binding site. However, these docking results do not establish the precise binding mode or confirm that p38α MAPK inhibition is responsible for the observed cellular effects. Nevertheless, its slightly less favorable docking score and the absence of the canonical Met109 hinge hydrogen bond observed for SB203580 suggest that Compound **29** may adopt a partially distinct binding mode.

As a future perspective, since no migration, invasion, pharmacokinetic, or *in vivo* experiments were conducted in the present work, the preliminary *in vitro* dual anti-cancer and anti-inflammatory profiles demonstrated by Compound **29** warrant further comprehensive validation. In addition, the lower IC_50_ values observed for the combination treatments should not be interpreted as definitive evidence of pharmacological synergy in the absence of a formal synergy analysis. To advance this scaffold along the drug discovery pipeline and establish its translational drug-likeness, subsequent research will focus on experimental ADMET profiling—encompassing aqueous solubility, microsomal stability, plasma stability, and membrane permeability—followed by preliminary *in vivo* pharmacokinetic and tolerability evaluations in appropriate animal models.

## Figures and Tables

**Table 1:** The chemical structures and substituent patterns of the synthesized thiazole-substituted benzoylpiperazine derivatives (**1**–**66**).

	Cpd. No	R	R′	Cpd. No	R	R′	Cpd. No	R	R′
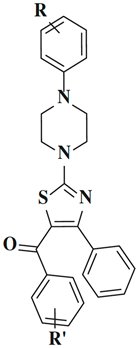	**1**	2-OCH_3_	H	**23**	4-OCH_3_	H	**45**	3-Cl	H
	**2**	2-OCH_3_	3-CH_3_	**24**	4-OCH_3_	3-CH_3_	**46**	3-Cl	3-CH_3_
	**3**	2-OCH_3_	4-CH_3_	**25**	4-OCH_3_	4-CH_3_	**47**	3-Cl	4-CH_3_
	**4**	2-OCH_3_	3-OCH_3_	**26**	4-OCH_3_	3-OCH_3_	**48**	3-Cl	3-OCH_3_
	**5**	2-OCH_3_	4-OCH_3_	**27**	4-OCH_3_	4-OCH_3_	**49**	3-Cl	4-OCH_3_
	**6**	2-OCH_3_	3-F	**28**	4-OCH_3_	3-F	**50**	3-Cl	3-F
	**7**	2-OCH_3_	4-F	**29**	4-OCH_3_	4-F	**51**	3-Cl	4-F
	**8**	2-OCH_3_	3-Cl	**30**	4-OCH_3_	3-Cl	**52**	3-Cl	3-Cl
	**9**	2-OCH_3_	4-Cl	**31**	4-OCH_3_	4-Cl	**53**	3-Cl	4-Cl
	**10**	2-OCH_3_	3-NO_2_	**32**	4-OCH_3_	3-NO_2_	**54**	3-Cl	3-NO_2_
	**11**	2-OCH_3_	4-NO_2_	**33**	4-OCH_3_	4-NO_2_	**55**	3-Cl	4-NO_2_
	**12**	3-OCH_3_	H	**34**	2-Cl	H	**56**	4-Cl	H
	**13**	3-OCH_3_	3-CH_3_	**35**	2-Cl	3-CH_3_	**57**	4-Cl	3-CH_3_
	**14**	3-OCH_3_	4-CH_3_	**36**	2-Cl	4-CH_3_	**58**	4-Cl	4-CH_3_
	**15**	3-OCH_3_	3-OCH_3_	**37**	2-Cl	3-OCH_3_	**59**	4-Cl	3-OCH_3_
	**16**	3-OCH_3_	4-OCH_3_	**38**	2-Cl	4-OCH_3_	**60**	4-Cl	4-OCH_3_
	**17**	3-OCH_3_	3-F	**39**	2-Cl	3-F	**61**	4-Cl	3-F
	**18**	3-OCH_3_	4-F	**40**	2-Cl	4-F	**62**	4-Cl	4-F
	**19**	3-OCH_3_	3-Cl	**41**	2-Cl	3-Cl	**63**	4-Cl	3-Cl
	**20**	3-OCH_3_	4-Cl	**42**	2-Cl	4-Cl	**64**	4-Cl	4-Cl
	**21**	3-OCH_3_	3-NO_2_	**43**	2-Cl	3-NO_2_	**65**	4-Cl	3-NO_2_
	**22**	3-OCH_3_	4-NO_2_	**44**	2-Cl	4-NO_2_	**66**	4-Cl	4-NO_2_

**Table 2:** IC_50_ values of compounds **1**–**66** and standards against HEK293, SHSY5Y, PC3, MCF7, and U87MG cell lines (µM).

Cpd. No	HEK293	SHSY5Y	PC3	MCF7	U87MG
**1**	73.90 ± 1.21	50.52 ± 1.44	58.83 ± 2.08	64.70 ± 2.33	79.45 ± 2.05
**2**	85.09 ± 1.33	17.87 ± 1.12	38.28 ± 1.98	45.24 ± 2.90	60.39 ± 2.03
**3**	88.27 ± 2.03	9.34 ± 0.99	27.80 ± 1.44	34.90 ± 1.44	50.51 ± 1.22
**4**	99.12 ± 1.88	19.20 ± 1.04	40.51 ± 1.65	43.88 ± 1.49	62.88 ± 1.53
**5**	98.53 ± 2.30	13.90 ± 0.98	25.99 ± 1.66	38.21 ± 1.37	54.23 ± 1.30
**6**	96.49 ± 2.04	7.99 ± 0.89	24.01 ± 1.33	30.01 ± 1.77	40.44 ± 1.45
**7**	95.01 ± 1.99	5.56 ± 0.97	14.32 ± 1.07	19.11 ± 1.42	33.41 ± 1.76
**8**	60.61 ± 1.20	38.90 ± 1.20	52.35 ± 2.40	58.32 ± 2.04	77.34 ± 1.19
**9**	62.99 ± 1.38	39.01 ± 1.10	57.50 ± 2.87	60.90 ± 2.22	73.77 ± 2.08
**10**	64.33 ± 1.76	11.39 ± 0.99	29.01 ± 1.46	33.70 ± 1.68	47.75 ± 1.55
**11**	54.90 ± 2.22	6.90 ± 0.97	17.40 ± 1.22	22.82 ± 1.08	36.90 ± 1.28
**12**	74.71 ± 1.82	48.57 ± 1.78	58.27 ± 2.06	58.39 ± 1.77	59.03 ± 1.39
**13**	77.89 ± 1.33	20.45 ± 1.65	37.29 ± 1.88	47.12 ± 1.11	64.90 ± 1.99
**14**	78.04 ± 1.98	10.22 ± 0.99	28.04 ± 1.21	33.84 ± 1.20	48.29 ± 1.21
**15**	86.92 ± 1.54	18.34 ± 1.11	41.90 ± 1.44	45.66 ± 1.87	67.04 ± 0.99
**16**	89.16 ± 1.99	14.83 ± 1.04	30.33 ± 2.05	38.21 ± 1.07	49.22 ± 1.08
**17**	93.38 ± 2.65	9.12 ± 0.99	22.46 ± 1.11	29.11 ± 1.08	42.46 ± 1.03
**18**	93.99 ± 1.82	4.99 ± 0.96	15.28 ± 1.06	22.80 ± 1.21	30.33 ± 1.15
**19**	61.88 ± 1.66	30.04 ± 1.35	47.43 ± 2.13	62.30 ± 2.28	67.64 ± 2.22
**20**	64.29 ± 2.01	33.47 ± 1.20	48.16 ± 2.21	55.39 ± 1.83	65.83 ± 1.53
**21**	64.80 ± 1.76	9.89 ± 0.99	26.01 ± 2.09	37.58 ± 1.76	44.80 ± 2.31
**22**	50.98 ± 1.23	6.37 ± 0.98	20.22 ± 1.73	24.27 ± 1.24	38.05 ± 1.98
**23**	70.98 ± 1.99	42.16 ± 1.11	55.62 ± 1.55	51.24 ± 2.45	68.96 ± 1.77
**24**	78.90 ± 2.30	10.55 ± 0.99	30.33 ± 1.36	33.21 ± 1.99	45.99 ± 1.45
**25**	80.01 ± 2.08	11.23 ± 1.03	27.70 ± 1.92	35.78 ± 1.43	48.72 ± 1.21
**26**	94.99 ± 2.99	8.90 ± 0.97	29.84 ± 1.67	36.49 ± 1.33	48.38 ± 1.74
**27**	97.21 ± 1.97	9.46 ± 0.98	26.36 ± 1.23	39.71 ± 1.97	46.09 ± 1.46
**28**	96.85 ± 1.80	8.53 ± 0.96	25.71 ± 1.37	27.80 ± 1.13	39.99 ± 1.93
**29**	98.10 ± 2.91	2.23 ± 0.95	12.99 ± 0.99	23.22 ± 1.20	32.07 ± 1.45
**30**	65.02 ± 1.56	27.31 ± 0.99	46.48 ± 1.46	50.99 ± 2.08	70.30 ± 2.50
**31**	62.87 ± 1.88	29.22 ± 1.05	49.33 ± 1.77	52.86 ± 2.22	76.49 ± 2.14
**32**	63.35 ± 1.57	13.04 ± 0.99	28.31 ± 0.99	36.31 ± 1.17	43.17 ± 1.77
**33**	54.89 ± 1.89	8.02 ± 0.98	15.99 ± 0.99	26.01 ± 1.40	30.88 ± 1.48
**34**	72.51 ± 2.07	50.60 ± 1.90	52.30 ± 2.03	60.82 ± 2.05	79.77 ± 1.90
**35**	>100	16.87 ± 1.02	39.02 ± 1.40	33.12 ± 1.38	58.39 ± 1.76
**36**	>100	17.99 ± 0.99	37.38 ± 1.93	30.99 ± 1.22	55.90 ± 2.07
**37**	>100	15.94 ± 0.99	41.28 ± 1.39	36.03 ± 1.39	57.22 ± 2.15
**38**	>100	19.05 ± 1.03	40.52 ± 1.45	35.31 ± 1.27	57.76 ± 2.30
**39**	>100	9.11 ± 0.98	23.88 ± 1.10	28.88 ± 1.11	40.44 ± 2.10
**40**	>100	10.03 ± 0.99	26.07 ± 1.27	29.02 ± 1.39	39.29 ± 2.04
**41**	61.59 ± 1.66	30.90 ± 1.24	49.93 ± 2.01	65.73 ± 2.77	77.15 ± 2.44
**42**	64.27 ± 1.45	34.02 ± 1.32	47.29 ± 1.99	62.39 ± 2.47	74.99 ± 2.17
**43**	55.01 ± 1.80	13.14 ± 1.06	30.33 ± 1.24	38.75 ± 2.11	45.83 ± 1.49
**44**	53.26 ± 1.53	6.22 ± 0.98	16.73 ± 0.99	21.18 ± 1.07	32.01 ± 1.37
**45**	75.03 ± 2.08	47.20 ± 2.01	51.70 ± 1.30	59.03 ± 2.05	77.23 ± 2.76
**46**	98.40 ± 2.69	20.22 ± 1.44	37.42 ± 1.20	37.04 ± 1.68	63.90 ± 2.02
**47**	97.99 ± 2.39	18.77 ± 1.09	39.74 ± 2.01	36.21 ± 1.45	67.32 ± 2.38
**48**	>100	20.34 ± 1.31	40.88 ± 2.35	37.99 ± 1.99	66.61 ± 1.85
**49**	>100	21.08 ± 1.02	42.69 ± 1.39	35.42 ± 1.34	65.30 ± 1.33
**50**	85.64 ± 2.07	8.64 ± 0.99	21.90 ± 1.02	30.11 ± 1.20	43.28 ± 1.40
**51**	87.30 ± 1.98	9.32 ± 0.98	23.99 ± 1.28	29.63 ± 1.22	40.43 ± 1.01
**52**	50.10 ± 1.35	27.48 ± 1.88	38.74 ± 1.40	44.70 ± 2.03	58.55 ± 2.41
**53**	53.99 ± 2.20	30.02 ± 1.46	40.92 ± 1.44	42.55 ± 2.03	56.99 ± 2.02
**54**	53.75 ± 1.39	13.49 ± 0.99	28.01 ± 1.32	35.30 ± 1.75	48.23 ± 1.54
**55**	44.67 ± 1.77	5.90 ± 0.95	16.28 ± 0.99	24.55 ± 1.08	36.84 ± 1.20
**56**	72.37 ± 2.06	49.82 ± 1.30	50.42 ± 2.01	57.80 ± 2.77	72.03 ± 2.26
**57**	86.90 ± 1.98	18.45 ± 1.08	37.13 ± 1.21	32.09 ± 1.54	62.98 ± 1.78
**58**	85.43 ± 2.43	12.76 ± 0.99	38.22 ± 1.44	33.70 ± 1.22	64.00 ± 1.55
**59**	94.99 ± 2.72	17.99 ± 1.02	36.20 ± 1.87	34.87 ± 2.02	61.80 ± 1.30
**60**	96.06 ± 1.87	10.98 ± 0.99	41.22 ± 1.66	37.42 ± 1.77	60.53 ± 1.26
**61**	85.88 ± 1.40	7.99 ± 0.98	22.55 ± 1.99	29.25 ± 1.05	38.77 ± 1.72
**62**	88.07 ± 2.92	4.98 ± 0.95	15.71 ± 0.99	18.99 ± 0.99	26.39 ± 1.55
**63**	54.02 ± 1.39	27.64 ± 1.07	40.44 ± 1.20	43.90 ± 2.19	60.12 ± 1.96
**64**	53.81 ± 1.70	28.11 ± 1.11	38.25 ± 1.65	45.21 ± 1.38	62.48 ± 1.44
**65**	45.01 ± 1.21	10.97 ± 0.99	27.26 ± 1.23	33.16 ± 1.67	47.00 ± 1.05
**66**	44.99 ± 2.09	4.99 ± 0.96	19.32 ± 1.20	27.05 ± 1.44	36.39 ± 1.11
lercanidipine	>100	35.03 ± 1.34	80.01 ± 2.11	98.76 ± 2.30	49.38 ± 1.27
cisplatin	3.11 ± 0.98	8.04 ± 0.99	7.11 ± 1.10	11.02 ± 1.64	18.99 ± 1.08

**Table 3:** SI values of compounds **1**–**66** and standards.

Cpd. No	IC_50(HEK293)_/IC_50(SHSY5Y)_	IC_50(HEK293)_/IC_50(PC3)_	IC_50(HEK293)_/IC_50(MCF7)_	IC_50(HEK293)_/IC_50(U87MG)_
**1**	1.46	1.25	1.25	1.14
**2**	4.76	2.22	1.88	1.40
**3**	9.45	3.17	2.52	1.74
**4**	5.16	2.44	2.25	1.57
**5**	7.08	3.79	2.57	1.81
**6**	12.07	4.01	3.21	2.38
**7**	17.08	6.63	4.97	2.84
**8**	1.55	1.15	1.03	0.78
**9**	1.61	1.09	1.03	0.85
**10**	5.64	2.21	1.90	1.34
**11**	7.95	3.15	2.40	1.48
**12**	1.53	1.28	1.27	1.26
**13**	3.80	2.08	1.65	1.20
**14**	7.63	2.78	2.30	1.61
**15**	4.73	2.07	1.90	1.29
**16**	6.01	2.93	2.33	1.81
**17**	10.23	4.15	3.20	2.19
**18**	18.83	6.15	4.12	3.09
**19**	2.05	1.30	0.99	0.91
**20**	1.92	1.33	1.16	0.97
**21**	6.55	2.49	1.72	1.44
**22**	8.00	2.52	2.10	1.33
**23**	1.68	1.27	1.38	1.02
**24**	7.47	2.60	2.37	1.71
**25**	7.12	2.88	2.23	1.64
**26**	10.67	3.18	2.60	1.96
**27**	10.27	3.68	2.44	2.10
**28**	11.35	3.76	3.48	2.42
**29**	43.99	7.55	4.22	3.05
**30**	2.38	1.39	1.27	0.92
**31**	2.15	1.27	1.18	0.82
**32**	4.85	2.23	1.74	1.46
**33**	6.84	3.43	2.11	1.77
**34**	1.43	1.38	1.19	0.90
**35**	5.92	2.56	3.01	1.71
**36**	5.55	2.67	3.22	1.78
**37**	6.27	2.42	2.77	1.74
**38**	5.24	2.46	2.83	1.73
**39**	10.97	4.18	3.46	2.47
**40**	9.97	3.83	3.44	2.54
**41**	1.99	1.23	0.93	0.79
**42**	1.88	1.35	1.03	0.85
**43**	4.18	1.81	1.41	1.20
**44**	8.56	3.18	2.51	1.66
**45**	1.58	1.45	1.27	0.97
**46**	4.86	2.62	2.65	1.53
**47**	5.22	2.46	2.70	1.45
**48**	4.92	2.44	2.63	1.50
**49**	4.74	2.34	2.82	1.53
**50**	9.91	3.91	2.84	1.97
**51**	9.36	3.63	2.94	2.15
**52**	1.82	1.29	1.12	0.85
**53**	1.79	1.31	1.26	0.94
**54**	3.98	1.91	1.52	1.11
**55**	7.57	2.74	1.81	1.21
**56**	1.45	1.43	1.25	1.00
**57**	4.71	2.34	1.50	1.37
**58**	6.69	2.23	2.53	1.33
**59**	5.28	2.62	2.72	1.53
**60**	8.74	2.33	2.56	1.58
**61**	10.74	3.80	2.93	2.21
**62**	17.68	5.60	4.63	3.33
**63**	1.95	1.33	1.23	0.89
**64**	1.91	1.40	1.19	0.86
**65**	4.10	1.65	1.35	0.95
**66**	1.00	2.75	1.66	1.23
lercanidipine	2.85	1.24	1.01	2.02
cisplatin	0.38	0.43	0.28	0.16

**Table 4:** Antiproliferative activity of Compound **29**, lercanidipine, cisplatin, and their fixed-ratio combinations, expressed as monotherapy and combination IC_50_ values.

Cell Lines	Monotherapy IC_50_ (μM)	Dual	Dual	Dual	Triple
	[Compound **29**/Lercanidipine/Cisplatin]	Compound **29** + Lercanidipine IC_50_ (μM)	Compound **29** + Cisplatin IC_50_ (μM)	Lercanidipine + Cisplatin IC_50_ (μM)	Compound **29** + Lercanidipine + Cisplatin IC_50_ (μM)
SHSY5Y	2.23 ± 0.95	1.31 ± 0.62	0.64 ± 0.55	12.97 ± 0.92	0.41 ± 0.53
	35.03 ± 1.34				
	8.04 ± 0.99				
PC3	12.99 ± 0.99	9.99 ± 0.78	5.41 ± 0.62	42.11 ± 0.91	2.82 ± 0.51
	80.01 ± 2.11				
	7.11 ± 1.10				
MCF7	23.22 ± 1.20	21.11 ± 1.45	8.29 ± 0.61	61.73 ± 1.02	5.66 ± 0.53
	98.76 ± 2.30				
	11.02 ± 1.64				
U87MG	32.07 ± 1.45	18.86 ± 1.21	11.06 ± 0.89	27.43 ± 0.99	5.17 ± 0.58
	49.38 ± 1.27				
	18.99 ± 1.08				

IC_50_ values represent mean ± SD from three independent biological experiments (*n* = 3).

## Data Availability

The original contributions presented in this study are included in the article. Further inquires can be directed to the corresponding author.

## Abbreviations

The following abbreviations are used in this manuscript:

CCBs	Calcium channel blockers
Ca^2+^	Intracellular calcium
DHP	Dihydropyridine
VEGFR	Vascular Endothelial Growth Factor Receptor
EGFR	Epidermal Growth Factor Receptor
MAPK	Mitogen Activated Protein Kinase
LPS	Lipopolysaccharide
MTT	3-(4,5-dimethylthiazol-2-yl)-2,5-diphenyltetrazolium bromide
DMSO	Dimethyl sulfoxide
PBS	Phosphate-buffered saline
FBS	Fetal bovine serum
SD	Standard deviation
VGCC	Voltage-gated calcium channel
